# The Impact of an Efflux Pump Inhibitor on the Activity of Free and Liposomal Antibiotics against *Pseudomonas aeruginosa*

**DOI:** 10.3390/pharmaceutics13040577

**Published:** 2021-04-18

**Authors:** Douweh Leyla Gbian, Abdelwahab Omri

**Affiliations:** Department of Chemistry and Biochemistry, The Novel Drug and Vaccine Delivery Systems Facility, Laurentian University, Sudbury, ON P3E 2C6, Canada; Douweh@laurentian.ca

**Keywords:** cystic fibrosis, *Pseudomonas aeruginosa*, liposomes, efflux pump inhibitor, PABN, aminoglycosides, macrolides

## Abstract

The eradication of *Pseudomonas aeruginosa* in cystic fibrosis patients has become continuously difficult due to its increased resistance to treatments. This study assessed the efficacy of free and liposomal gentamicin and erythromycin, combined with Phenylalanine arginine beta-naphthylamide (PABN), a broad-spectrum efflux pump inhibitor, against *P. aeruginosa* isolates. Liposomes were prepared and characterized for their sizes and encapsulation efficiencies. The antimicrobial activities of formulations were determined by the microbroth dilution method. Their activity on *P. aeruginosa* biofilms was assessed, and the effect of sub-inhibitory concentrations on bacterial virulence factors, quorum sensing (QS) signals and bacterial motility was also evaluated. The average diameters of liposomes were 562.67 ± 33.74 nm for gentamicin and 3086.35 ± 553.95 nm for erythromycin, with encapsulation efficiencies of 13.89 ± 1.54% and 51.58 ± 2.84%, respectively. Liposomes and PABN combinations potentiated antibiotics by reducing minimum inhibitory and bactericidal concentrations by 4–32 fold overall. The formulations significantly inhibited biofilm formation and differentially attenuated virulence factor production as well as motility. Unexpectedly, QS signal production was not affected by treatments. Taken together, the results indicate that PABN shows potential as an adjuvant of liposomal macrolides and aminoglycosides in the management of lung infections in cystic fibrosis patients.

## 1. Introduction

*Pseudomonas aeruginosa* is an opportunistic Gram-negative bacterium and the principal pathogen found in the lungs of cystic fibrosis (CF) patients [1,2]. Chronic and persistent pulmonary infections caused by *P. aeruginosa* lead to progressive lung damage, and eventually respiratory failure and death [3]. They are the leading cause of death in CF [4]. Aminoglycosides and macrolides are commonly prescribed for the management of *P. aeruginosa* infections in CF, as they inhibit protein synthesis in the bacteria [2,5,6,7,8]. Moreover, macrolides such as azithromycin are recommended for CF patients because they reduce pulmonary exacerbations over long periods [9,10]. However, due to the bacteria’s increased resistance to clinically acceptable levels of antibiotics and the associated toxicity of macrolides and aminoglycosides at those high concentrations, it is crucial to develop new ways to revitalize those drugs [2,11,12,13]. Poor drug penetration is a major issue behind bacterial resistance to antibiotics. It can be attributable to reduced membrane permeability to antibiotics [14] and to the expression of efflux pumps which thwart the activity of antimicrobials by inducing their expulsion from the cell [15,16,17,18].

The main efflux systems with the highest clinical significance in *P. aeruginosa* are MexAB-OprM, MexCD-OprJ, MexEF-OprN, and MexXY-OprM pumps, which belong to the resistance nodulation cell division family and export metabolites, antibiotics and even quorum sensing (QS) molecules [15,19]. The MexAB-OprM pump transports beta-lactams, macrolides, tetracyclines [20], and 3-oxo-dodecanoyl homoserine lactone (3OC_12_-HSL, QS signal) [21] among others, while the MexXY-OprM pump exports aminoglycosides, fluoroquinolones, tetracyclines and macrolides [22,23,24].

QS is used by bacteria to coordinate group behaviors at high cell density via the production of signaling molecules called autoinducers [25]. *P. aeruginosa* has three main interconnected QS systems organized hierarchically, namely the LasI/R system at the top, followed by the RhlI/R and the *Pseudomonas* Quinolone Signal (PQS) systems, respectively [26,27]. For QS to occur, autoinducers must be expressed extracellularly and detected by neighboring cells. N-butanoyl-homoserine lactone (C_4_-HSL) signals produced by the Rhl system freely diffuse out of the cells, while 3OC_12_-HSL and PQS signals produced by Las and PQS, respectively, need to be exported by membrane transporters to cross the outer membrane [21,25]. *P. aeruginosa* upregulates virulence factors such as pyocyanin, proteases, motilities and forms robust biofilms through QS, causing destructive infections and inflammations [28,29,30]. Pyocyanin is a redox-active toxin that plays a crucial role in the establishment of *P. aeruginosa*’s infection, while proteases and lipases target and degrade the host’s proteins and lipids to facilitate the bacterium’s invasion [29]. Pyoverdine, on the other hand, is a siderophore used for scavenging iron, vital for bacterial growth and virulence [31]. A summary of *P. aeruginosa*’s main efflux pumps, QS systems and virulence factors along with their functions is provided in Table 1 below. Biofilms are communities of bacteria attached to a surface, protected by an exopolysaccharide matrix that can be 10–1000 times more resistant to antibiotics than planktonic bacteria [32]. Furthermore, recent studies have suggested a positive connection between biofilms and efflux pumps [33]. However, the exact mechanism behind this is not fully elucidated.

Combining efflux pump inhibitors with antibiotics could therefore represent a good strategy to bypass efflux resistance [41]. One such compound is Phenylalanine-Arginine Naphthylamide (PABN), a broad-spectrum competitive efflux pump inhibitor thought to behave as a substrate of efflux pumps by binding to their transporter domains [42]. PABN acts directly on efflux pumps without affecting the proton gradient and the electrical potential across the inner cell membrane [43]. It is reported to potentiate in vitro activity of various anti-pseudomonal drugs including fluoroquinolones, beta-lactams and aminoglycosides against multidrug resistant *P. aeruginosa* strains [44] and to even inhibit QS and virulence factors [43].

Antibiotics’ encapsulation into liposomes can also be used to overcome poor drug penetration. Liposomes are spherical lipid vesicles of one or more lipid bilayers that serve as carriers for hydrophilic, lipophilic and amphiphilic compounds [45]. They protect drugs from undesired metabolic breakdown, increase their accumulation at the target site and reduce their toxicity, as less product is needed for therapeutic effect [46,47]. Arikayce^®^ (Insmed), for instance, is a liposomal preparation of amikacin recently approved by the FDA (Food and Drug Administration, USA), used against *Mycobacterium avium* complex and *P. aeruginosa*, showing superior efficacy than its free counterpart [48,49]. Furthermore, previous work from our group demonstrated that liposomal antibiotics showed increased antimicrobial activities against resistant *P. aeruginosa* strains isolated from CF patients [47,50,51,52].

In the present study, we prepared liposomal gentamicin (GEN) and erythromycin (ERY), and their antimicrobial activity in combination with PABN was assessed against *P. aeruginosa* strains. Indeed, their impact on bacteria’s biofilms, virulence factors and QS signal production as well as motilities was evaluated.

## 2. Materials and Methods

### 2.1. Chemicals and Media

Gentamicin, agarose, chloroform and casamino acids were obtained from Fisher Scientific (Ottawa, ON, Canada). Erythromycin was purchased from Caledon Laboratories LTD (Georgetown, WA, Canada). Phenylalanine-Arginine-β-Naphthylamide, cholesterol and Triton X-100 were purchased from Sigma Aldrich (Oakville, ON, Canada). The compound 2-Nitrophenyl-β-d-galactopyranoside was obtained from Thermo Fisher Scientific (Ottawa, ON, Canada). DPPC (1,2-Dipalmitoyl-*sn*-glycero-3-phosphocholine) was obtained from Avanti Polar Lipids Inc. (Alabaster, AL, USA). Mueller–Hinton broth (MHB) and Lysogeny broth (LB) and agar were purchased from Becton Dickinson (Francklin Lakes, NJ, USA) and Becton Dickinson Microbiology Systems (Oakville, ON, Canada), respectively. ABt medium and Z buffer were prepared as described previously [47,51].

### 2.2. Bacterial Strains

PA01 was a generous gift from Dr R.E.W Hancock (University of British Columbia, Vancouver, BC, Canada), and the clinical strain PA11 was obtained from the Memorial Hospital’s Clinical Microbiology Laboratory (Sudbury, ON, Canada). *Staphylococcus aureus* (ATCC 29213) and *Bacillus subtilis* (ATCC 6633) strains purchased from PML Microbiologicals (Mississauga, ON, Canada) were used as indicator organisms for gentamicin and erythromycin, respectively [53]. All strains were stored at −80 °C in MHB supplemented with 10% glycerol (*v*/*v*) in a −86 °C ULT Freezer, Thermo Forma. The *Agrobacterium tumefaciens* strain A136 (pCF218)(pCF372) (Ti-) kindly donated by Dr Fuqua (Indiana University, Bloomington, IN, USA) was used as the biosensor strain and cultured in LB broth at 30 °C for the detection of acyl homoserine lactones (AHLs) [47].

### 2.3. Preparation of Liposomes

Gentamicin and erythromycin were encapsulated into liposomes composed of DPPC (0.11382 g/mL) and cholesterol (0.01 g/mL) at a molar ratio of 6:1 (DPPC to cholesterol), using the dehydration–rehydration vesicle (DRV) method as previously described [52]. Erythromycin was directly mixed with the lipids in the organic solvent for a final concentration of 20 mg/mL after rehydration. The lipid layer was rehydrated with a solution of 3 mg/mL of gentamicin or PBS (Phosphate-Buffered Saline) for erythromycin. Lipid suspensions were vortexed for 5 min and sonicated for 2 × 15 min (cycles of 45 s ON and 10 s OFF) in an ultrasonic dismembrator bath (FS20H; Fisher Scientific, Ottawa, ON, Canada) with an amplitude of 45 Hz (Model 500, Fisher Scientific). Lipid suspensions were divided into aliquots of 1 mL and frozen for 15 min, then placed overnight in a freeze-dry system (model 77540, Labanco Corporation, Kansas City, MO, USA). Powdered formulations obtained were stored in a freezer at 0 °C until use. Liposomes were rehydrated as previously described [52]. One hundred microliters of PBS was added to the powders and the mixtures were vortexed and incubated for 5 min at 40 °C. This step was repeated three times and a final volume of 700 µL of PBS was added. The unencapsulated drug was removed with three rounds of washing with PBS using a centrifuge (16,000 *g* for 15 min at 4 °C). The Submicron Nicomp particle sizer Model 270 (Nicomp, Santa Barbara, CA, USA) was used to measure the average particle size of liposomes and determine the polydispersity index as reported earlier [52].

### 2.4. Microbiological Assay for the Measurement of Gentamicin and Erythromycin in Liposomes

To measure the concentrations of antibiotics incorporated into liposomes, a microbiological agar diffusion assay with indicator strains was performed as previously reported [52]. The quantifiable limit for both antibiotics was 7.81 × 10^−3^ mg/mL. Standard curves linearity extended over 0.00781–4 mg/mL and gave correlation coefficients >0.99. Concentrations obtained were the means of three independent experiments performed in triplicate.

### 2.5. Determination of Encapsulation Efficiency

Encapsulation efficiencies (EE) of liposomal antibiotics were determined as the percentage of antibiotics entrapped in liposomes with respect to the initial amount used, with the following Equation (1) [53]:

(1) $$\mathrm{Encapsulation}\;\mathrm{efficiency}\;(\%)=\frac{\mathrm{Concentration}\;\mathrm{of}\;\mathrm{encapsulated}\;\mathrm{drug}\;\times 100}{\mathrm{Initial}\;\mathrm{drug}\;\mathrm{concentration}}$$

### 2.6. Determination of Minimum Inhibitory Concentrations (MICs) and Minimum Bactericidal Concentrations (MBCs)

MICs and MBCs of bacteria exposed to free and liposomal gentamicin and erythromycin with and without PABN (at 25 mg/L) were determined using the microbroth dilution technique as reported previously [50,54]. Results were taken from three separate experiments. Reductions of 2 fold or more with PABN were considered significant [44].

### 2.7. Minimum Biofilm Eradication Concentration (MBEC)

Biofilms of PA01 and PA11 adjusted to 0.5 McFarland standard (1.5 × 10^8^ CFU/mL) were grown for 72–96 h in MHB, in an MBEC^TM^ plate (CBD-Innovotech, Edmonton, AB, Canada) as recommended [55]. After incubation, the peg lid with biofilms was rinsed in a fresh 96-well plate filled with PBS, transferred to another plate containing serial dilutions of antibiotics combined with PABN at 25 mg/L and incubated for 24 h, at 37 °C, 110 rpm in the shaking incubator. Control wells were filled with MHB instead. After incubation, the peg lid was rinsed with PBS for a few seconds and placed in a recovery plate, a fresh 96-well plate with 200 µL of MHB per well and biofilms were sonicated for 30 min in an ultrasonic dismembrator bath. The recovery plate was incubated for 24 h at 37 °C, 110 rpm and the MBEC was determined as the smallest concentration of antibiotics to eradicate biofilms. Reductions of 2 fold or more with PABN were considered significant [44].

### 2.8. Effects of Sub-Inhibitory Concentrations of Free and Liposomal Gentamicin and Erythromycin on the Growth of P. aeruginosa

Bacterial solutions of *P. aeruginosa* equivalent to 2 times 0.5 McFarland standard or optical density at λ = 600 nm (OD600), absorbance 0.26 in MHB were prepared, modified from previous reports [56] and exposed to equal volumes of antibiotics at 1/2 MIC, 1/4 MIC, 1/8 MIC, 1/16 MIC and 1/32 MIC (when necessary). The study was performed for 7 h as described earlier [57]. Results were taken from three separate experiments.

### 2.9. Virulence Factor Assays

PA01 and PA11 standardized to 0.5 McFarland standard in LB broth supplemented with an equal volume of sub-inhibitory concentrations of antibiotics combined with PABN were incubated for 24 h at 200 rpm, 37 °C in a shaking incubator [58]. PBS was used instead for positive controls. After incubation, samples were centrifuged at 12,000 rpm, 4 °C for 20 min and the supernatant was filter sterilized for further use.

#### 2.9.1. Protease Assay

The assay was carried out with 1.25% (*v*/*v*) skimmed milk and filtered supernatants as previously described [59]. Protease activity = OD_600_ of skimmed milk − OD_600_ of each sample. Experiments were performed three times in triplicate.

#### 2.9.2. Pyocyanin and Pyoverdine Assays

Pyocyanin was extracted from supernatants with chloroform (1:3 *v*/*v* chloroform to supernatant) and quantified spectrophotometrically at λ = 520 nm as described earlier [57,58]. Pyoverdine in the aqueous layer was removed and the absorbance measured at 405 nm. Experiments were performed three times for pyocyanin and three times in triplicate for pyoverdine.

#### 2.9.3. Lipase Assay

The assay was performed as reported earlier [52]. A 0.6 mL aliquot of filtered supernatants of bacteria was mixed in a 15 mL centrifuge tube with 0.6 mL of Tween^®^ 80 in Tris-buffered saline (10% *v*/*v*), 0.1 mL of CaCl_2_ (1 M), and 1.2 mL of H_2_O and incubated for 24 h, at 37 °C and 200 rpm (Innova 4000 Incubator Shaker, New Brunswick Scientific, NJ, USA). In the presence of lipase, Tween is broken down and binds to calcium, which precipitates and can be quantified spectrophotometrically at λ = 400 nm. Experiments were performed three times in triplicate.

### 2.10. Beta-Galactosidase Activity Assay

AHL production levels from *P. aeruginosa* exposed to free and liposomal antibiotics at sub-inhibitory concentrations with and without PABN were evaluated with the reporter strain *A. tumefaciens* (A136) as previously described [52]. Briefly, 4 mL of the reporter strain was mixed with 1 mL of supernatant and incubated at 30 °C in a water bath for 5 h. The bacterial cell densities of the samples at 600 nm were then measured before centrifugation. The supernatant was discarded, and the pellet was resuspended in an equal volume of Z buffer, as described previously. The cells were permeabilized with 200 µL of chloroform and 100 µL of 0.1% SDS, before the addition of *o*-nitro phenol-β-d-galactopyranoside (4 mg/mL in PBS). After the development of a deep yellow color, the reaction was stopped with 1 mL of 1 M Na_2_CO_3_ and the absorbances of the samples were measured at 420 and 550 nm. Miller units of β-galactosidase activity were calculated as follows: 1000 × [*A*_420_ − (1.75 × *A*_550_)]/(time × volume × *A*_600_) [60].

### 2.11. Motility Studies

Motility studies were slightly modified from other investigators [51,58]. Standardized bacteria grown overnight (2 μL) were inoculated onto agar plates containing sub-inhibitory concentrations of free or liposomal antibiotics with and without PABN. Twitching, swarming and swimming plates were prepared as described earlier [51]. Plates were incubated in a CO_2_ incubator at 37 °C. After 18 h, swimming and swarming diameters were measured while twitching diameters were measured after 24 h. Experiments were performed three times in triplicate.

### 2.12. Data Analysis

Data are represented as mean ± SEM (standard error of the mean) of three independent experiments. Comparison between groups was achieved by one-way analysis of variance (ANOVA) with a Tukey–Kramer Multiple Comparisons test with GraphPad prism (GraphPad Software Inc., San Diego, CA, USA, version 8.4.3). Probability values of * *p* ˂ 0.05, ** *p* ˂ 0.01, *** *p* ˂ 0.001 and **** *p* ˂ 0.0001 were considered statistically significant.

## 3. Results

### 3.1. Liposomal Antibiotics Characterisations

The encapsulation efficiency (EE) of liposomal GEN was 13.89 ± 1.545% and the concentration entrapped was 0.42 ± 0.046 mg/mL (Table 2). On the other hand, the EE of ERY was 51.58 ± 2.846% with an entrapped concentration of 10.32 ± 0.571 mg/mL. The average diameters of liposomal GEN and ERY were 562.67 ± 33.74 nm and 3086.35 ± 553.95 nm, respectively. The polydispersity index, which is a measure of size distribution, comprised between 0.0 (homogeneous) and 1.0 (heterogeneous) ranged from 0.6 ± 0.12 to 0.7 ± 0.11 for ERY and GEN, respectively.

### 3.2. Determination of MICs, MBCs and MBECs

Liposomal antibiotics combined with PABN reduced MICs and MBCs in both strains by 4–32 fold as presented in Table 3. For instance, the MIC of PA11 was 256 mg/L for free GEN, 32 mg/L for liposomal GEN and 8 mg/L for liposomal GEN with PABN. Similar trends were observed for the MBCs. Liposomal formulations with PABN also eradicated biofilms and strongly reduced MBECs by 8–32 fold for GEN and 2–16 fold for ERY in both strains (Table 4). However, in PA11 no significant changes in MBEC were noticed after the addition of PABN to liposomal ERY. Additionally, the MIC values of PABN alone were 256 mg/L and 512 mg/L in PA01 and PA11, respectively, and the MBC in both strains was of 512 mg/L (not shown here). The MICs and MBCs for quality control laboratory strains were within the acceptable limits established by CLSI, Clinical and Laboratory Standards Institute (formerly NCCLS, National Committee for Clinical Laboratory Standards), as previously found in our group [61]. The liposomes containing PBS (control) had no antibacterial activity. Likewise, the combination of empty liposomes with free drug had no additive effect on the antibacterial activity of GEN and ERY.

### 3.3. Effects of Sub-Inhibitory Concentrations of Free and Liposomal Antibiotics on the Growth of P. aeruginosa Strains

**Figure 1:**
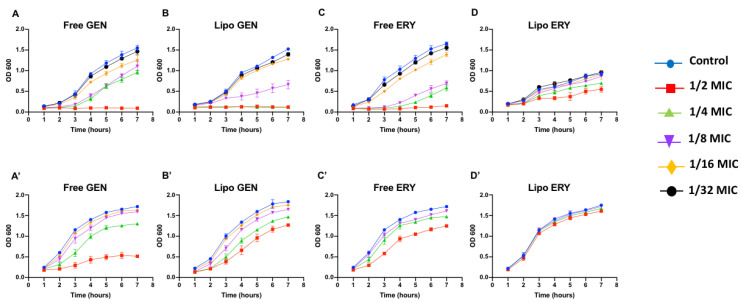
The effects of sub-inhibitory concentrations of free and liposomal gentamicin and erythromycin on the growth of PA01 and PA11 at 1/2, 1/4, 1/8, 1/16 and 1/32 the MIC. Shown are PA01 with free gentamicin (**A**), liposomal gentamicin (**B**), free erythromycin (**C**), liposomal erythromycin (**D**) and PA11 with free gentamicin (**A’**), liposomal gentamicin (**B’**), free erythromycin (**C’**) and liposomal erythromycin (**D’**).

Sub-inhibitory concentrations of 1/16 and 1/32 the MIC did not seem to significantly inhibit PA01 growth (Figure 1A–D). In PA11, 1/16 MIC for both free and liposomal antibiotic tests did not affect bacterial growth (Figure 1A’–D’). Concentrations of 1/16 and 1/32 the MIC were therefore chosen to study the effects of sub-inhibitory concentrations of antibiotics on virulence factors, motility and the production of QS molecules in both strains.

### 3.4. Effect of Antibiotics and PABN on Bacterial Virulence Factors

**Figure 2:**
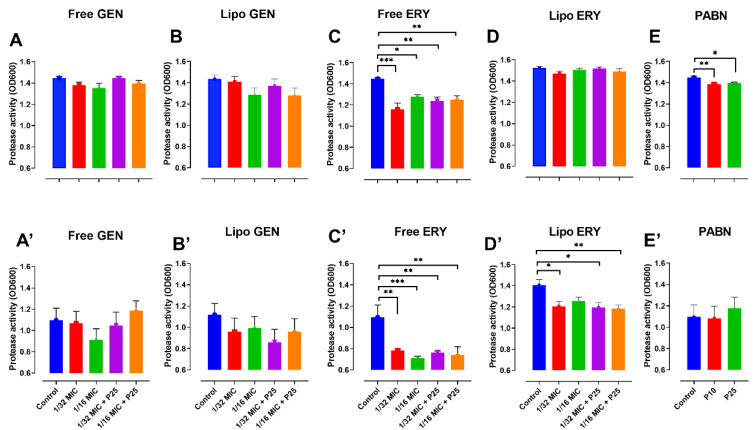
Effects of sub-inhibitory concentrations of free and liposomal gentamicin and erythromycin in the presence and absence of PABN on protease levels in PA01 and PA11. Shown are PA01 with free gentamicin (**A**), liposomal gentamicin (**B**), free erythromycin (**C**), liposomal erythromycin (**D**), PABN (**E**) and PA11 with free gentamicin (**A’**), liposomal gentamicin (**B’**), free erythromycin (**C’**), liposomal erythromycin (**D’**) and PABN (**E’**). The results represent the mean ± SEM of three independent experiments performed in triplicate. *p* values were considered significant when compared with the control and between groups: ***, *p* < 0.001; **, *p* < 0.01; *, *p* < 0.05. P10 and P25 correspond to PABN used at 10 and 25 mg/L, respectively.

Protease was significantly reduced by free erythromycin with and without PABN (*p* < 0.001) in both strains (Figure 2C,C’), liposomal erythromycin at 1/16 MIC with PABN in PA11 (*p* < 0.01) (Figure 2D’) and by PABN alone in PA01 (*p* < 0.01) (Figure 2E).

**Figure 3:**
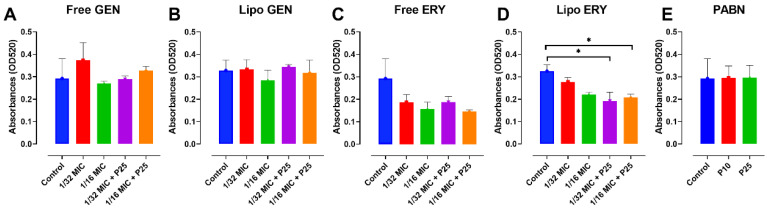
Effects of sub-inhibitory concentrations of free and liposomal gentamicin and erythromycin in the presence and absence of PABN on pyocyanin levels in PA01. Shown are PA01 with free gentamicin (**A**), liposomal gentamicin (**B**), free erythromycin (**C**), liposomal erythromycin (**D**) and PABN (**E**). The results represent the mean ± SEM of three independent experiments. *p* values were considered significant when compared with the control and between groups: *, *p* < 0.05. P10 and P25 correspond to PABN used at 10 and 25 mg/L, respectively.

Only liposomal erythromycin combined with PABN seemed effective in significantly reducing pyocyanin production in PA01 (*p* < 0.05), as shown in Figure 3D. The reduction induced by free erythromycin (Figure 3C) seems considerable, but its significance appears to be prevented by variabilities in the control samples. Similarly, even though in Figure 3D the effects of 1/16 MIC and 1/16 MIC + P25 look identical, their respective values of 0.220 and 0.207 explain why the latter is significant while the other is not. No significant changes in the production of pyocyanin were noticed between the samples with and without PABN. It should be noted that the pyocyanin assay was only performed in PA01, as the strain PA11 did not appear to produce the compound.

**Figure 4:**
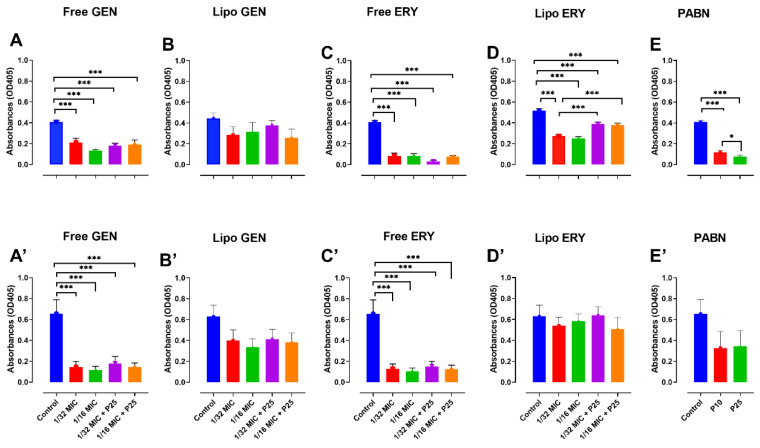
Effects of sub-inhibitory concentrations of free and liposomal gentamicin and erythromycin in the presence and absence of PABN on pyoverdine levels in PA01 and PA11. Shown are PA01 with free gentamicin (**A**), liposomal gentamicin (**B**), free erythromycin (**C**), liposomal erythromycin (**D**), PABN (**E**) and PA11 with free gentamicin (**A’**), liposomal gentamicin (**B’**), free erythromycin (**C’**), liposomal erythromycin (**D’**) and PABN (**E’**). The results represent the mean ± SEM of three independent experiments performed in triplicate. *p* values were considered significant when compared with the control and between groups: ***, *p* < 0.001; *, *p* < 0.05. P10 and P25 correspond to PABN used at 10 and 25 mg/L, respectively.

Pyoverdine in PA01 was greatly reduced by free and liposomal erythromycin (*p* < 0.001) and free gentamicin (*p* < 0.001) with and without PABN and by PABN alone (*p* < 0.001) (Figure 4A,C–E). In PA11, pyoverdine was significantly lowered by free antibiotics with and without PABN (*p* < 0.001) (Figure 4A’,C’).

**Figure 5:**
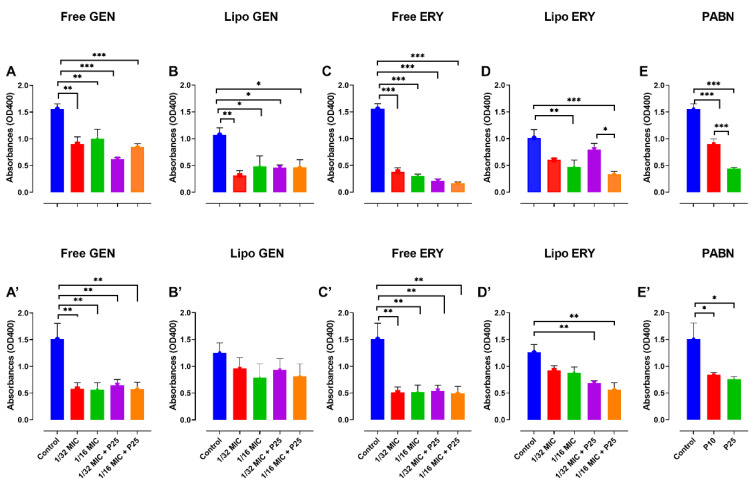
Effects of sub-inhibitory concentrations of free and liposomal gentamicin and erythromycin in the presence and absence of PABN on lipase levels in PA01 and PA11. Shown are PA01 with free gentamicin (**A**), liposomal gentamicin (**B**), free erythromycin (**C**), liposomal erythromycin (**D**), PABN (**E**) and PA11 with free gentamicin (**A’**), liposomal gentamicin (**B’**), free erythromycin (**C’**), liposomal erythromycin (**D’**) and PABN (**E’**). The results represent the mean ± SEM of three independent experiments performed in triplicate. *p* values were considered significant when compared with the control and between groups: ***, *p* < 0.001; **, *p* < 0.01; *, *p* < 0.05. P10 and P25 correspond to PABN used at 10 and 25 mg/L, respectively.

Finally, lipase production was significantly diminished by all our treatments in PA01 (*p* < 0.05, *p* < 0.01 and *p* < 0.001) (Figure 5A–E). In some instances, this effect was greater when PABN was added. Lipase production was also significantly reduced in PA11 by free and liposomal erythromycin (*p* < 0.01) and by free gentamicin (*p* < 0.01) in Figure 5A’,C’–E’. Furthermore, PABN alone was highly effective at reducing lipase in both strains (*p* < 0.001 in PA01 and *p* < 0.05 in PA11).

### 3.5. Assessment of Quorum-Sensing Signal Production through a Beta-Galactosidase Assay

**Figure 6:**
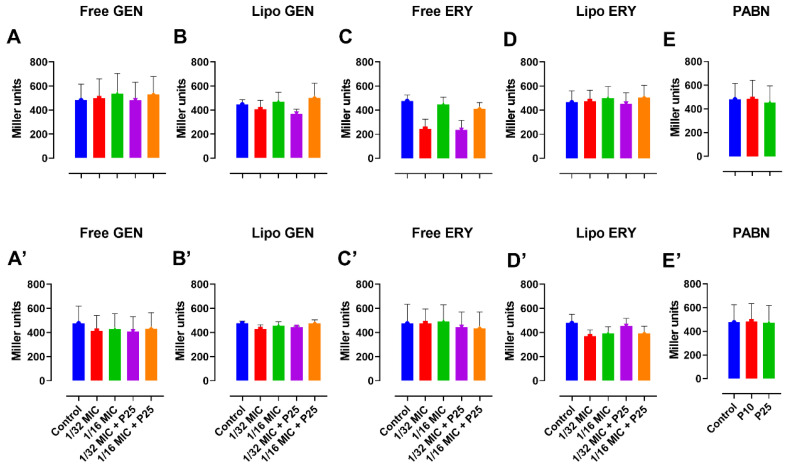
Effects of sub-inhibitory concentrations of free and liposomal gentamicin and erythromycin in the presence and absence of PABN on quorum sensing signals in PA01 and PA11. Shown are PA01 with free gentamicin (**A**), free erythromycin (**B**), liposomal gentamicin (**C**), liposomal erythromycin (**D**), PABN (**E**) and PA11 with free gentamicin (**A’**), free erythromycin (**B’**), liposomal gentamicin (**C’**), liposomal erythromycin (**D’**) and PABN (**E’**). The results represent the mean ± SEM of three independent experiments performed in triplicate. P10 and P25 correspond to PABN used at 10 and 25 mg/L, respectively.

There were no statistically significant reductions observed in the levels of AHLs produced in both PA01 and PA11 from the β-galactosidase assay, as shown in Figure 6A–E’.

### 3.6. Effect of Antibiotics and PABN on Bacterial Motility

**Figure 7:**
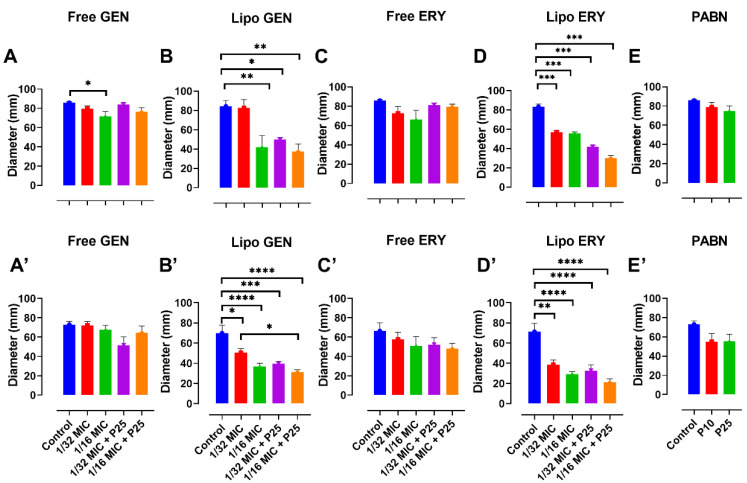
Impact of sub-inhibitory concentrations of free and liposomal gentamicin and erythromycin with PABN on PA01 and PA11 twitching motility. Twitching was examined with free and liposomal antibiotics at 1/16 and 1/32 the MIC in the presence and absence of PABN (10 and 25 mg/L). Shown are PA01 with free gentamicin (**A**), liposomal gentamicin (**B**), free erythromycin (**C**), liposomal erythromycin (**D**), PABN (**E**) and PA11 with free gentamicin (**A’**), liposomal gentamicin (**B’**), free erythromycin (**C’**), liposomal erythromycin (**D’**) and PABN (**E’**). The results are represented as the mean ± SEM of three independent experiments in triplicates. *p* values were considered significant compared with the control and between groups: ****, *p* < 0.0001; ***, *p* < 0.001; **, *p* < 0.01; *, *p* < 0.05. P10 and P25 correspond to PABN used at 10 and 25 mg/L, respectively.

**Figure 8:**
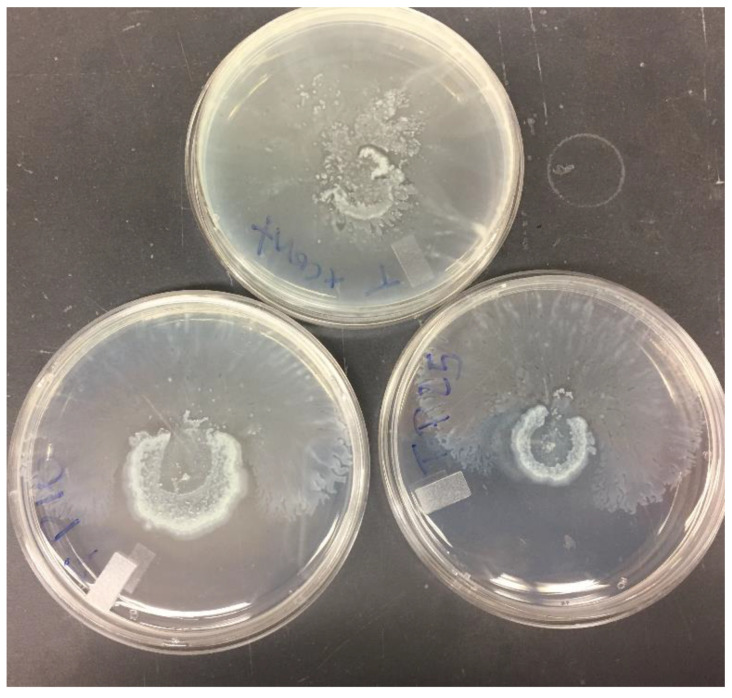
Twitching motility of PA11 with PABN at 10 and 25 mg/L. This picture shows an example of twitching motility observed with one of our strains. For consistency, the largest value for the diameters was used during the motility studies.

In PA01, twitching was significantly reduced at 1/16 MIC with PABN by liposomal gentamicin (*p* < 0.01) and liposomal erythromycin (*p* < 0.001) (Figure 7B,D). An example of the twitching motility is shown in Figure 8.

**Figure 9:**
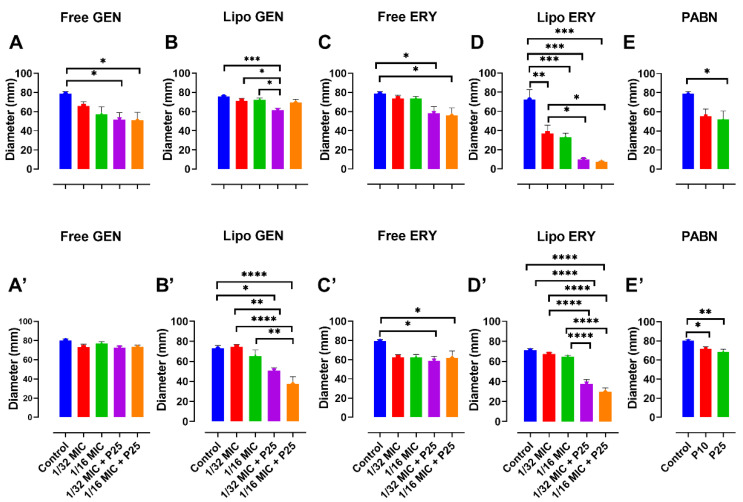
Impact of sub-inhibitory concentrations of free and liposomal gentamicin and erythromycin with PABN on PA01 and PA11 swarming motility. Swarming was examined with free and liposomal antibiotics at 1/16 and 1/32 the MIC in the presence and absence of PABN (10 and 25 mg/L). Shown are PA01 with free gentamicin (**A**), liposomal gentamicin (**B**), free erythromycin (**C**), liposomal erythromycin (**D**), PABN (**E**) and PA11 with free gentamicin (**A’**), liposomal gentamicin (**B’**), free erythromycin (**C’**), liposomal erythromycin (**D’**) and PABN (**E’**). The results are represented as the mean ± SEM of three independent experiments in triplicates. *p* values were considered significant compared with the control and between groups: ****, *p* < 0.0001; ***, *p* < 0.001; **, *p* < 0.01; *, *p* < 0.05. P10 and P25 correspond to PABN used at 10 and 25 mg/L, respectively.

**Figure 10:**
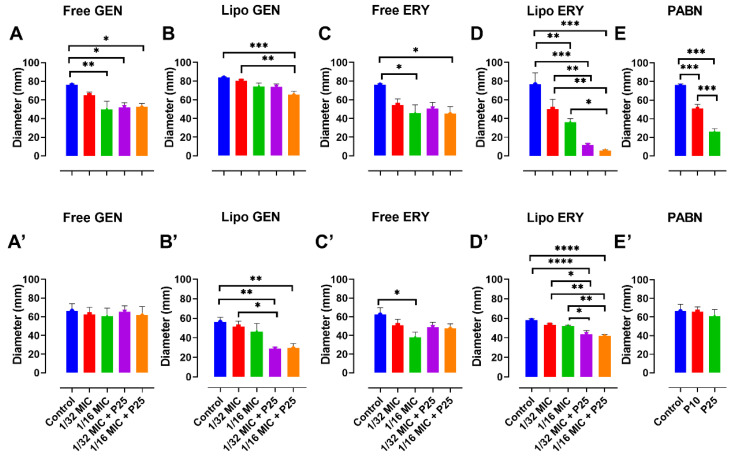
Impact of sub-inhibitory concentrations of free and liposomal gentamicin and erythromycin with PABN on PA01 and PA11 swimming motility. Swimming was examined with free and liposomal antibiotics at 1/16 and 1/32 the MIC in the presence and absence of PABN (10 and 25 mg/L). Shown are PA01 with free gentamicin (**A**), liposomal gentamicin (**B**), free erythromycin (**C**), liposomal erythromycin (**D**), PABN (**E**) and PA11 with free gentamicin (**A’**), liposomal gentamicin (**B’**), free erythromycin (**C’**), liposomal erythromycin (**D’**) and PABN (**E’**). The results are represented as the mean ± SEM of three independent experiments in triplicates. *p* values were considered significant compared with the control and between groups: ****, *p* < 0.0001; ***, *p* < 0.001; **, *p* < 0.01; *, *p* < 0.05. P10 and P25 correspond to PABN used at 10 and 25 mg/L, respectively.

Swarming and swimming were also considerably inhibited by all formulations (*p* < 0.05, *p* < 0.01 and *p* < 0.001) (Figure 9A–E and Figure 10A–E). In PA11, only liposomal gentamicin and erythromycin significantly inhibited twitching (*p* < 0.0001) at 1/16 MIC with and without PABN (Figure 7B’,D’). Swarming was strongly inhibited by liposomal antibiotics at 1/16 MIC (*p* < 0.0001), free erythromycin with PABN (*p* < 0.05) and PABN alone (*p* < 0.05 and *p* < 0.01) (Figure 9B’–E’). Finally, swimming was significantly reduced by liposomal gentamicin and erythromycin with PABN (*p* < 0.01 and *p* < 0.0001, respectively) and by free erythromycin at 1/16 MIC (*p* < 0.05) (Figure 10B’–D’).

## 4. Discussion

In the present study, liposomal gentamicin and erythromycin composed of DPPC-cholesterol were prepared by the DRV method in an attempt to increase their antimicrobial activity against resistant strains of *P. aeruginosa*. The polydispersity indexes indicate that our liposomal samples were fairly heterogeneous overall. Liposomal gentamicin showed superior EE than previous studies that reported values of 4.51% with DMPC-cholesterol and 1.8% with DPPC-cholesterol, respectively, for gentamicin [50,62]. Similarly, our EE for erythromycin was higher than earlier studies with erythromycin (32.06%) [53] and other macrolides such as azithromycin (23.08%) [52] and clarithromycin (15.96%) [51]. The direct dissolution of erythromycin in the organic solution with lipids due to its lipophilic nature, combined with an increased sonication time in our method (5 min vs. 30 min), might be behind these results. In fact, increased sonication time was shown to enhance drug EEs [63].

Liposomal formulations showed enhanced inhibitory and bactericidal activities against *P. aeruginosa* in comparison to free drugs. Indeed, bacteria went from resistant to intermediate or susceptible to treatments. Similar observations were reported by earlier studies with liposomal aminoglycosides and macrolides, showing that liposomes increased bacterial killing of free antibiotics [18,50,62,64,65]. Liposomes’ increased activity was proposed to be the result of their fusion, disruption of the bacterial cell membrane and the subsequent intracellular uptake of their content [61,64]. When added, PABN further decreased MICs and MBCs especially in PA11, suggesting that efflux pumps could be one of the main resistance mechanisms in this strain. The ability of PABN to inhibit efflux pumps in *P. aeruginosa* was evaluated by Lamers and coworkers as well through a fluorescence assay, with a fluorescent probe that is also an efflux substrate. The probe’s fluorescence is only observed when it is bound to nucleic acids inside cells. PABN caused significant increases in fluorescence by 23–32% at 25 mg/L, indicating its significant inhibition of efflux in those strains [44]. We expect similar reductions in efflux activity in our strains treated with PABN, although further studies are needed to confirm this. Liposomes and PABN considerably reduced MBECs in both strains. Our results extend the findings of Ye et al. and Bandara et al., who found that tobramycin/clarithromycin proliposomes and liposomal ciprofloxacin, respectively, significantly eradicated *P. aeruginosa* biofilms when compared to free drugs [65,66]. Furthermore, Halwani et al. demonstrated that liposomal gentamicin co-encapsulated with gallium completely eradicated *P. aeruginosa* biofilms in vitro [54]. Similarly, Ferrer et al. reported that efflux pump inhibitors such as PABN combined with membrane permeabilizing peptides render *P. aeruginosa* strains that are overexpressing MexAB-OprM pumps more sensitive to antibiotics [42]. This strategy is interesting as it is believed to considerably reduce the associated toxicity of EPIs such as PABN. The minimum biofilm eradication concentration (MBIC) is the lowest concentration of an antimicrobial substance that induces no time dependent increase in the mean number of biofilm viable cells. It is commonly used to assess the inhibitory effects of formulations on biofilms [67]. It could be useful for future studies to determine the MBIC to fully and accurately determine the effects of our formulations on *P. aeruginosa* biofilms.

Virulence factors were reduced by most of our formulations to various extents, especially by free and liposomal erythromycin. This might account for the role of macrolides in the attenuation of *P. aeruginosa* inflammation at sub-inhibitory concentrations through the inhibition of virulence factors among other mechanisms [68,69]. Khan et al. also showed that free aminoglycosides including gentamicin significantly inhibited virulence factors such as pyoverdine, protease and pyocyanin in *P. aeruginosa* strains [7,70]. Earlier studies demonstrated an increased inhibitory activity of liposomal antibiotics on protease, elastase, lipase and chitinase production [47,51,52]. In contrast, our liposomal formulations did not appear to show superior activity overall against *P. aeruginosa* virulence factors. However, even in those instances liposomes are still of interest since studies have demonstrated their reduced toxicity and enhanced distribution in vivo [18]. Our results compare well with El-Shaer et al., who found that PABN alone reduced virulence factors in *P. aeruginosa* [43]. The results also extend the findings of Giordano et al., who indicated that PABN has a profound impact on *P. aeruginosa* transcriptome and affects virulence factors differentially [71].

Unexpectedly, none of our treatments showed significant reductions in the production of AHLs in both strains, even though virulence factors controlled by QS seemed to have been reduced. Similar studies report a reduction in QS signal levels in *P. aeruginosa* by sub-inhibitory concentrations of antibiotics or adjuvants [72,73,74,75]. El-Shaer et al., for example, reported a reduction in QS signals with PABN alone in *P. aeruginosa* strains isolated from urinary tract and wound infections [43]. However, this effect was not observed for all their strains, as C_4_-HSL levels in wound isolates were unchanged and the level of reduction reported varied significantly between strains. This suggests a strain-dependant activity of PABN which might explain the differences in our results. It is also possible our treatments affected the detection (signal/receptor interaction) and/or the transport of autoinducers as well as reduced the expression of virulence genes. For instance, Khan et al. recently showed through an in silico docking analysis that aminoglycosides interact with *P. aeruginosa* QS receptor LasR. They proposed this to be a mechanism by which they inhibit QS associated virulence factors in the bacterium, as it prevents the binding of LasR receptor to 3OC_12_-HSL signals [7]. Furthermore, Burr et al. reported that sub-inhibitory concentrations of erythromycin strongly inhibited the expression of *P. aeruginosa* QS genes such as LasR and PqsA in non-CF bronchiectasis airways [69]. Similarly, El-Shaer and coworkers showed that PABN reduced the expression of QS genes such as LasI/R (with more specificity for LasR), RhlI/R and PqsA/R as well as virulence genes, suggesting again an activity of PABN on *P. aeruginosa* transcription, beyond efflux inhibition [43]. Interestingly, Giordano et al. found that PABN enhanced the transcription of *qteE*, a gene coding for a protein that inhibits the activity of 3OC_12_-HSL receptor, LasR [71]. Numerous lines of evidence also demonstrated the involvement of efflux pumps in the transport of some autoinducers. Indeed, the MexAB-OprM pump is implicated in the efflux of 3OC_12_-HSL and QS-regulated factors are affected by its activity [21]. Furthermore, MexEF-OprN and MexGHI-OpmD pumps were shown to export precursors of the PQS signals, facilitate QS and bacterial growth and to promote virulence [76,77,78]. It is therefore possible that by inhibiting efflux pumps, PABN could have affected the transport of some autoinducers. This could result in less signals being transported and detected, and therefore lead to a reduced expression of QS related genes like virulence factors and motility [43].

In *P. aeruginosa*, twitching is modulated by type IV pili and is evident on solid surfaces [79], while swarming is a coordinated group movement on semi-solid surfaces that requires both flagella and type IV pili, and as such, it is regulated by QS [80]. Swimming, on the other hand, occurs in a liquid environment and also requires the use of flagella [79,81]. All those motilities play a determining role in bacterial attachment, colonization and their ability to cause widespread infections [7]. Motilities in bacteria were significantly reduced by our formulations to various extents. However, liposomal antibiotics proved to be more efficacious, especially in PA11. It was found that PABN alone inhibited motilities in *P. aeruginosa* [43]. Our results are also supported by previous studies which showed that erythromycin inhibited swarming in *P. aeruginosa* and that azithromycin and gentamicin inhibited twitching and swarming in PA01 [82,83]. The former study explained that macrolides inhibit flagellin expression in the bacteria, which is needed for the production of flagella, used in swarming and swimming.

## 5. Conclusions

Adjuvant therapy is an interesting strategy to revitalize the activity of old antibiotics. Indeed, liposomal gentamicin and erythromycin combined with PABN proved efficacious overall in inhibiting *P. aeruginosa* growth, eradicating biofilms and reducing the production of virulence factors and motility, even though the production of QS autoinducers did not appear to be affected. This suggests a possible impairment of the detection and/or transport of QS signals by our formulations, which should be confirmed through molecular studies. Furthermore, in vivo studies are needed to fully appreciate the impact of our treatments on the course of an infection in biological systems. Liposomal gentamicin and erythromycin with PABN therefore show potential in the management of *P. aeruginosa* infections in cystic fibrosis patients.

## Figures and Tables

**Table 1:** Summary of *P. aeruginosa*’s main efflux pumps, quorum sensing (QS) systems and virulence factors.

Efflux Pumps	Substrates	QS Systems (Molecules)	Function	Virulence Factors	Function
MexAB-OprM	Beta-lactams, macrolides, tetracyclines [20], and 3-oxo-dodecanoyl homoserine lactone (3OC_12_-HSL, QS signal) [21]	LasI/R (3OC_12_-HSL)	Regulates elastase, protease, exotoxin A, biofilm formation and induces PQS and Rhl systems [34,35]	Protease	Immune invasion and host tissue damage [7,29]
MexXY-OprM	Aminoglycosides, fluoroquinolones, tetracyclines and macrolides [22,23,24]	RhlI/R (C_4_-HSL)	Regulates the production of pyocyanin, rhamnolipids, elastase and hydrogen cyanide [36]	Pyocyanin	Induces oxidative stress, neutrophil apoptosis, inhibits ciliary beating in the airways, and causes cytotoxicity [37,38]
MexCD-OprJ	Macrolides, cephalosporins, fluoroquinolones, tetracyclines and organic solvents [39]	PQS ^1^ (PQS signal)	Regulates the expression of pyoverdine, pyocyanin, rhamnolipids and the RhlI/R system [35]	Pyoverdine	Iron scavenging, vital for pathogenesis. Sequestrates iron from host, which is used for biofilm formation [40]
MexEF-OprN	Chloramphenicol, tetracycline, fluoroquinolones, HHQ ^2^ (QS signal) [39]			Lipase	Degrades lipids in the host [29]

^1^*Pseudomonas* quinolone signal, ^2^ 4-hydroxy-2-heptylquinoline.

**Table 2:** Characterization of liposomal gentamicin and erythromycin.

Liposomal Antibiotics	Size (nm)	Polydispersity Index	Encapsulation Efficiency (%)	Concentration (mg/mL)
Gentamicin	562.67 ± 33.74	0.7 ± 0.11	13.89 ± 1.545	0.42 ± 0.046
Erythromycin	3086.35 ± 553.95	0.6 ± 0.12	51.58 ± 2.846	10.32 ± 0.571

**Table 3:** Free and liposomal antibiotics susceptibility of *P. aeruginosa* isolates.

Strains	MIC (mg/L) (MBC *(mg/L))				MIC (mg/L) (MBC * (mg/L))			
	Free GEN		Lipo GEN		Free ERY		Lipo ERY	
	−PABN	+PABN	−PABN	+PABN	−PABN	+PABN	−PABN	+PABN
PA 01	8 (32)	8 (16)	2 (4)	1 (2)	512 (512)	256 (256)	128 (128)	128 (128)
PA 11	256 (1024)	32 (128)	32 (128)	8 (32)	1024 (1024)	512 (512)	128 (256)	64(256)

* Minimum bactericidal concentrations are shown in parentheses.

**Table 4:** Efficacy of free and liposomal antibiotics against biofilms of *P. aeruginosa* isolates.

Strains	MBEC (mg/L)							
	Free GEN		Lipo GEN		Free ERY		Lipo ERY	
	−PABN	+PABN	−PABN	+PABN	−PABN	+PABN	−PABN	+PABN
PA 01	64	16	4	2	1024	1024	128	64
PA 11	1024	128	256	128	1024	512	512	512

## Data Availability

Not applicable.
